# Gut Microbiota Dysbiosis Is a Crucial Player for the Poor Outcomes for COVID-19 in Elderly, Diabetic and Hypertensive Patients

**DOI:** 10.3389/fmed.2021.644751

**Published:** 2021-08-11

**Authors:** Nathalia Santos Magalhães, Wilson Savino, Patrícia Machado Rodrigues Silva, Marco Aurélio Martins, Vinicius Frias Carvalho

**Affiliations:** ^1^Laboratory of Inflammation, Oswaldo Cruz Institute, Oswaldo Cruz Foundation (Fiocruz), Rio de Janeiro, Brazil; ^2^Laboratory on Thymus Research, Oswaldo Cruz Institute, Oswaldo Cruz Foundation (Fiocruz), Rio de Janeiro, Brazil; ^3^National Institute of Science and Technology on Neuroimmunomodulation (INCT-NIM), Oswaldo Cruz Institute, Oswaldo Cruz Foundation (Fiocruz), Rio de Janeiro, Brazil; ^4^Rio de Janeiro Research Network on Neuroinflammation (RENEURIN), Oswaldo Cruz Institute, Oswaldo Cruz Foundation (Fiocruz), Rio de Janeiro, Brazil

**Keywords:** COVID19, aging, diabetes, gut microbiota, hypertension, SARS-CoV-2

## Abstract

A new infectious disease, named COVID-19, caused by the coronavirus associated to severe acute respiratory syndrome (SARS-CoV-2) has become pandemic in 2020. The three most common pre-existing comorbidities associated with COVID-19-related death are elderly, diabetic, and hypertensive people. A common factor among these risk groups for the outcome of death in patients infected with SARS-CoV-2 is dysbiosis, with an increase in the proportion of bacteria with a pro-inflammatory profile. Due to this dysbiosis, elderly, diabetic, and hypertensive people present a higher propensity to mount an inflammatory environment in the gut with poor immune editing, culminating in a weakness of the intestinal permeability barrier and high bacterial product translocation to the bloodstream. This scenario culminates in a low-grade, persistent, and systemic inflammation. In this context, we propose here that high circulating levels of bacterial products, like lipopolysaccharide (LPS), can potentiate the SARS-CoV-2-induced cytokines, including IL-6, being crucial for development of the cytokine storm in the severe form of the disease. A better understanding on the possible correlation between gut dysbiosis and poor outcomes observed in elderly, diabetic, and hypertensive people can be useful for the development of new therapeutic strategies based on modulation of the gut microbiota.

## Introduction

In early December 2019, a new infectious disease, caused by the coronavirus associated to severe acute respiratory syndrome (SARS-CoV-2), emerged in Wuhan, China (1). The disease caused by this infection, COVID-19, spread very rapidly in many other countries reaching pandemic proportions (2, 3). By 24 May 2021, there were 166,814,851 individuals diagnosed with COVID-19, including 3,458,905 fatal cases, as shown in the WHO data center (4). In severe COVID-19 patients, 93% of deaths result from respiratory failure caused by acute respiratory distress syndrome (ARDS). Besides, the storm of cytokines and symptoms of sepsis, with failure of some vital organs, including heart and kidney, derived by the primary viral infection and/or secondary infections were observed in 70% of fatal cases (5). No specific effective therapeutics are so far available for COVID-19 and the management of the disease includes physical distancing, mask wearing, supportive medical care, and vaccines (4). Herein, we propose a role of gut dysbiosis in the worse prognosis of COVID-19 in elderly people and in patients with *Diabetes mellitus* (DM) or hypertension.

## COVID-19 and Gut Microbiota

The human microbiota is made up of microorganisms, including bacteria, fungi, archaea, viruses, and protozoa, that colonize particular locations of the human body such as skin, as well as respiratory and gastrointestinal tracts (6, 7). The gut microbiota refers specifically to a complex bacterial community situated in the gastrointestinal tract (8). Although approximately 40% of patients infected with SARS-CoV-2 showed a high concentration of viral genetic material in the anal swab, and various patients reported nausea, vomiting, and diarrhea (9, 10) little has been so far discussed on the role of the gut in the pathophysiology of COVID-19, especially envisioning microbiota as being responsible for the greatest risk factor to develop the severe form of the disease.

It is well known that the membrane angiotensin I converting enzyme 2 (ACE2) is the pathway of entry into the target cells (11). Human mature enterocytes located in the small intestine express membrane ACE2, and SARS-CoV-2 is able to infect those cells in a process facilitated by TMPRSS2 and TMPRSS4 proteases (12). The infection of enterocytes with SARS-CoV-2 may promote a significant reduction of enteric ACE2 integrity/functionality. The decrease of ACE2 expression leads to an upregulation of other renin-angiotensin system components, including angiotensin (Ang) II (13). Remarkably, increased Ang II levels can modify gut microbial composition and metabolomics in a sex-specific manner (14). In addition, the SARS-CoV-2 infection-induced reduction of ACE2 function may also culminate in gut dysbiosis through a decrease in the mTOR-mediated synthesis of AMPs independently of RAS (15).

The possibility that SARS-CoV-2 infection of enterocytes modify gut microbiota is supported by the fact that some patients with COVID-19 present intestinal dysbiosis (16, 17). There is evidence that hospitalized COVID-19 patients exhibit a significant reduction in gut microbiome diversity with depletion of beneficial bacterial symbionts and enrichment of opportunistic pathogens, including *Actinomyces, Rothia*, and *Streptococcus* (17, 18). Patients infected with SARS-CoV-2 also showed a decrease in the relative abundance of *Faecalibacterium prausnitzii* and *Bifidobacterium bifidum*, which are bacteria responsible for the production of butyrate (17, 19). Butyrate is a short-chain fatty acid (SCFA) that influences both the proliferation and differentiation of epithelial intestinal cells, by enhancing the renewal and integrity of the epithelial barrier function (20). Moreover, patients undergoing allogeneic hematopoietic cell transplantation showing greater abundance of butyrate-producing bacteria have five-fold protection against the development of viral lower respiratory tract infection (21).

Interestingly, there are several pathologies in which the gut microbiota is modified and in some of them a direct relationship has been found with the severity of COVID-19, including elderly, diabetes, hypertension, obesity, periodontitis, and kidney diseases, as summarized in Table 1. Among these conditions, aging, diabetes, and hypertension stand out, since they are the main cause of COVID-19-related death (95–99). Yet, before getting into this point, it seemed worthwhile to discuss basic aspects of the gut microbiota, as well as the dysbiosis seen in aging and disease, particularly diabetes and hypertension.

**Table 1:** Summary of the alterations in the gut microbiota, gut immune cells, blood and gut cytokine profiles in main groups at risk for COVID-19.

**Condition**	**Species**	**Gut microbiota**	**Gut imune cells**	**Gut cytokines**	**Blood cytokines**	**Ref**
Aging	Murine model	↑*Prevotella sp*. ↓*Lachnospiraceae* ↓*Akkermansia sp*. ↓*Lactobacillus sp*.	↓ Th1 ↑ Th17 ↑ Treg	↑ IL-4 ↓ IL-10 ↓TGF-β	↑ IL-1β ↓ IL-2 ↑ IL-6 ↑ IL-8 ↑ IL-13 ↑ IL-17 ↑ TNF	(22–28)
	Human	↑*Clostridium difficile* ↑*Enterobacter spp*. *↑ Enterobacteriaceae*. *↑ Eubacterium sp*. *↑ Staphylococcus spp* *↑Streptococcus spp*. *↓ Akkermansia sp*. *↓ Bifidobacterium sp*. *↓ Faecalibacterium sp*. *↓ Lactobacillus spp*	↓ Th17	↑ IL-6	↑ IL-1β ↓ IL-2 ↓ IL-4 ↑ IL-6 ↑ IL10 ↑ IL-17 ↑ IL-18 ↑ TGF-β ↑ TNFα	(23, 29–43)
Diabetes	Murine model	*↓ Faecalibacterium sp*. ↓*Akkermansia muciniphila*	↓ Th2 ↑ Th17 ↓ Treg	↑ IL-10 ↓ IL-18 ↑ IL-17 ↑ IL-23	↑ IL-1β ↑ IL-6	(44–50)
	Human	↑*Bacteroides* ↑*Clostridium sp*. ↓*Akkermansia muciniphila* *↓ Eubacterium rectale* *↓ Faecalibacterium sp*. ↓*Roseburia sp*.	-	-	↑ IL-10 ↑ IL-17 ↓ IL-18 ↑ IL-23	(51–58)
Hypertension	Murine model	↑*Prevotella* ↑*Streptococcus spp*. ↓*Lactobacillus spp* ↓*Bifidobacterium sp*. ↓*Roseburia*	↑ Th17	↑ IL-1β↓ IL-6 ↓ IL-7 ↓TGF-β1 ↑ TNF-α	↑ IL-1β ↑ IL-6 ↑ IL8 ↑ IL-17 ↑ TNF-α	(59–67)
	Human	↑*Klebsiella*, ↑*Desulfovibrio* ↑*Prevotella* ↓*Blautia*, ↓*Butyrivibrio* ↓*Clostridium* ↓*Enterococcus* ↓*Faecalibacterium* ↓*Oscillbacter* ↓*Roseburia* ↓*Bifidobacterium* ↓*Lactobacillus*	-	-	↑ IL-6 ↑ TNF	(59, 60, 66, 68)
Obesity	Murine model	↑*Mollicutes* ↓*Akkermansia muciniphila* ↓*Bacteroides* ↓*Bacteroides thetaiotaomicron* ↓*Bifidobacterium* ↓*Enterobacteriale* ↓*Lactobacillus* ↓*Prevotella*	↑ Th1 ↑ Th17 ↓ Treg	↑ IL-1β ↓ IL-10 ↓ IL-17 ↑ IL-18 ↓ IL-22 ↑ TNFα	↑ IL-1β ↑ IL-6 ↑ TNF α	(69–72)
	Human	↑*Clostridium sp*. ↑*Eubacterium* ↓*Bifidobacteria* *↓ Faecalibacterium sp*. ↓*Bacteroides* ↓*Lactobacillus sp*. ↓*Akkermansia muciniphila*	↑ Th1 ↓ Treg	-	↑ IL-1 ↑ IL-5 ↑ IL-6 ↑ IL-10 ↑ IL-12 ↑ IL-13 ↑ IL-23 ↑ IL-36	(69, 72–77)
				↑ IFN-γ ↑ TNF-α	
Periodontitis	Murine model	↑*Bacteroidetes* ↑*Prevotella* *↓ Lactobacillus spp*	↑ IL-1β ↑ Th17	↑ IL-1β ↑ IL-6 ↑ IL-12b ↑ IL-17c ↑ TNFα ↑ TGF-β	↑ IL-1β ↑ IL-6 ↑ TNFα	(78–82)
	Human	↑*Enterobacteriaceae* ↑*Eubacteriaceae* *↓ Faecalibacterium sp*.	↑ Th17	↑ IL-17↑ IFNγ	↑ IL-1 ↑ IL-6 ↑ IL-17 ↑ IL-22 ↑ INFγ ↑ TNFα	(81, 83, 84)
Kidney Disease	Murine model	↑*Bifidobacterium* *↓ Lactobacillaceae* ↓ Prevotellaceae	-	↑ IL-1β ↑ IL-6 ↑ IL-12b ↑ IL-17a ↑ TNFα ↑ IFNγ	↑ IL-1β ↑ IL-5 ↑ IL-6 ↑ IL-10 ↑ IL-12 ↑ IFNγ ↑ TNFα	(85–89)
	Human	↑*Clostridium* ↑*Enterobacteriaceae* ↑*Streptococcaceae* ↑*Streptococcus* *↓ Roseburia* *↓ Faecalibacterium sp*. *↓ Lactobacillus* *↓ Prevotellaceae*	-	-	↑ IL-1β ↑ IL-6 ↑ TNFα	(90–94)

## Aging and Gut Microbiota

Aging is usually accompanied by a progressive decline of physiological functions determined by (epi) genetic, stochastic, and environmental processes (100). The elderly population has an increasing tendency to multimorbidity, fragility and disability. One of the biological systems most compromised by senility is the gastrointestinal tract (101). Along with aging, there is a degeneration of the enteric nervous system (ENS), alteration of intestinal motility, and changes in the intestinal mucous barrier, decreasing the defense function and favoring the development of gastrointestinal disorders (101, 102).

A mutual characteristic of aging in tissues and aging-related diseases is the *inflammaging*, which is the low-grade, persistent and systemic inflammation, even in the absence of infection, culminating in tissue degeneration and chronic diseases (101, 103). In addition, other hallmarks of immunosenescence are represented by a decrease in the capacity to respond to new antigens and the accumulation of memory T cells (103, 104). In aging, the gut dysbiosis leads, at least partly, to immune dysfunction, culminating in a more inflammatory environment with poor immune editing (29, 105). It is important to know that although the gut microbiota does not age its profile changes during aging. Furthermore, the maintenance of a “youthful” or “healthy” gut microbiota architecture throughout aging may postpone or limit immunosenescence (22).

During aging, the gut microbiota is characterized by an increase in the expression of proteolytic genes and a decrease in saccharolytic ones leading to the growth of pathogens, which in turn intensify inflammation (29). The most striking change in the microbiota of elderly individuals is the change in the relative proportions of Firmicutes and Bacteroidetes; the elderly having a higher proportion of Bacteroidetes, while in young adults the Firmicutes prevail (30). Moreover, the production of anti-inflammatory factors by the microbiota of elderly individuals is reduced, including butyrate (29). All these alterations observed in the gut microbiota during aging enhance a more pro-inflammatory environment, contributing to *inflammaging*.

Aging-associated gut dysbiosis induces a weakening of the intestinal barrier (102). Therefore, it is possible to observe high levels of bacterial products in the bloodstream such as LPS (31, 103), which could lead to an increase in the production of pro-inflammatory mediators. Indeed, elderly people have a rise in the amount of circulating cytokines as well as a decrease in the lymphocyte response, natural killer cells, and phagocytic activity (32, 103). Furthermore, aging animals have increased inflammatory cytokines in the plasma and an augmentation in the intestinal permeability compared to young animals (33). This pro-inflammatory status seems to be related causally to the microbiota profile, since aged GF animals do not present *inflammaging* status. In addition, when both aged and young GF animals received the microbiota from aging wild type (WT) animals, they exhibited an increase in the circulating contents of inflammatory cytokines and intestinal permeability. Aging animals also showed an increase in the LPS-induced inflammatory cell infiltration and IL-6 levels compared to young animals, indicating the development of ARDS that is one of the most prevalent morbidities associated with aging. Nevertheless, old GF mice presented less LPS-evoked inflammatory infiltrates in the lungs compared to WT animals (33). Therefore, the microbiota of aging animals is important to the development of *inflammaging*.

## Diabetes and Gut Microbiota

Diabetes Mellitus is a group of metabolic diseases characterized by hyperglycemia. Usually, DM is classified as type 1 and type 2 and related to low production and failure of insulin action, respectively (106). Nevertheless, this simple subdivision is not accurate, because it does not take into account the intermediate forms of DM with overlapping features. The “double diabetes” or type 1.5 diabetes is a disease with metabolic characteristics of type 2 DM with autoantibodies for β-cells typical of type 1 DM (107). Another intermediate form of DM is the Latent Autoimmune Diabetes in Adults (LADA), which shares autoimmune destruction of β-cells and insulin resistance, although to a lesser extent than type 1 DM (108). The hyperglycemia noted in diabetic patients is accompanied by the presence of cytokines such as IL-1β, IL-6, and TNF-α, characterizing a low-grade inflammation status (109).

A common change in all types of DM patients is the dysbiosis (110, 111). Although there is a controversy about which bacterial phyla is altered in the gut microbiota of diabetic patients, it is a consensus that the relationship between Firmicutes and Bacteroidetes is unbalanced in these patients (51, 112, 113). Besides, diabetic animals treated with probiotics containing the *Lactobacillus rhamnosus* NCDC17 improved the parameters regarding oral glucose tolerance test and led to an increase in plasma insulin, together with decreased the inflammatory cytokines IL-6 and TNF in the epididymal fat (114). Therefore, the absence or excessive proliferation of some bacteria could be one of the mechanisms of intestinal barrier dysfunction observed in diabetic models, leading to increased permeability of bacterial content to the bloodstream, as LPS (110). Replacement with *Faecalibacterium sp*. in diabetic animals improved the intestinal barrier integrity and circulating LPS levels (115).

Interestingly, the gut microbiota of non-obese diabetic mice changed before the onset of diabetes (52). Alterations observed included reduction of bacteria abundance and diversity, and one of the most affected groups was the butyrate-producing bacteria (53). Butyrate regulates the permeability of the intestinal barrier by inducing mucin production and decreasing the transit of bacteria, oxidative stress, as well as local and systemic inflammation (54). Accordingly, the increased permeability of the intestinal barrier observed in diabetic patients can be attributed, at least partly, to the reduction of butyrate-producing bacteria (55). Thus, it is plausible to think that butyrate replacement in diabetic patients, through direct administration or ingestion of prebiotics, may reduce intestinal permeability and low-grade inflammation triggered by gut microbiota products translocated into the bloodstream.

## Hypertension and Gut Microbiota

Hypertension is a progressive cardiovascular syndrome whose early markers are usually present even before the sustained increase of blood pressure (BP). The progression of hypertension may be represented as stages 1, 2, and 3. In stage 1, patients present occasional or intermittent BP elevations, early cardiovascular disease, and no target organ disease. In stage 2, patients exhibit sustained BP elevations or progressive cardiovascular disease and early signs of target organ disease. In stage 3, the patients show marked and sustained BP elevations or advanced cardiovascular disease and overtly present target organ disease (116). Unfortunately, despite advances in awareness about lifestyle improvements, new therapies, and intensive medical interventions, around a third of hypertensive patients do not obtain control of BP when prescribed three or more antihypertensive drugs, presenting the so-called “treatment-resistant” hypertension (59).

Although the etiology of hypertension seems to depend on both genetic and environmental factors, the exact cause remains unknown. Several pieces of evidence suggest that hypertension can result from intestinal dysbiosis. For instance, treatment with antibiotics produces an increase in BP, indicating the participation of gut microbiota in the control of BP (60). Furthermore, GF mice showed lower BP as compared to conventional ones and present attenuation of BP increase in response to infused angiotensin II (61). Also, metabolites of gut microbiota are involved in the control of BP, including trimethylamine N-oxide, hydrogen sulfate, and SCFAs (117).

Causative evidence for the role of gut dysbiosis in the genesis of hypertension came since transfection of dysbiotic fecal samples from hypertensive patients to GF mice raised BP in the recipients (22). A study carried out in pre-hypertensive and hypertensive patients detected a lower richness and diversity of the intestinal microbiota as compared to healthy individuals. Hypertensive patients presented an increase of gram-negative groups and an elevation of the ratio between Firmicutes and Bacteroidetes (22, 34, 35).

Gut microbiota and their metabolites reduce the epithelium barrier integrity during hypertension, and this is linked to the downregulation of tight junction protein expression (118, 119). Hypertensive rats also presented a higher intestinal permeability to trimethylamine (TMA), a microbiota metabolite precursor of trimethylamine N-oxide, which is a marker of cardiovascular mortality. Furthermore, spontaneously hypertensive rats (SHR) showed suppression of components of T cell receptor signaling cascade in the colonic epithelium compared to Wistar Kyoto (WKY) normotensive rats, including glycoprotein CD3 gamma chain and lymphocyte cytosolic protein 2 (Lcp2). SHR animals also presented a decrease in the expression of IL-6, IL-7, and TGF-β1 in the colonic epithelium, related to marked lower production of alkaline phosphatase in the intestinal epithelial cells (120). Together, these alterations in the colonic epithelium of SHRs characterize changes in the gut immune response and epithelial layer in hypertension.

It is well known that one of the major triggers of hypertension is the imbalanced diet with high salt content (121, 122). Such high salt environment induces Th17 cells (62, 123), which are pro-inflammatory; being also involved with the development of hypertension (63, 68). Mice and humans exposed to a high salt challenge showed depletion of *Lactobacillus spp*. in the gut microbiome along with the rise of Th17 cells and BP (35), indicating an association of Th17 cells produced by gut microbiota and the generation of hypertension. Of note, an increase in pro-inflammatory cytokines was also reported in hypertensive rats (64). In particular, IL-6 is a central cytokine in the regulation of BP, since it is responsive to angiotensin II to raise BP regardless of baseline values (65). Furthermore, a study carried out in hypertensive patients found an increase in pro-inflammatory cytokines in peripheral blood samples associated with changes in the profile of intestinal microbiota (124).

## Can Gut Microbiota Dysbiosis Be Important To SARS-CoV-2-Induced Immune Hyperresponsiveness and SARS Development in Elderly, Diabetic, and Hypertensive Individuals?

The main groups at risk for the COVID-19-related death are aging, DM, and hypertension. These conditions have a key point in common, which is dysbiosis that results in high intestinal permeability, translocation of bacterial contents to the bloodstream, and the development of basal inflammation. Therefore, a central question arises from this observation: can dysbiosis and the consequent pro-inflammatory status be critical for development of COVID-19 severity in aging, DM, and hypertensive individuals, similar to SARS and hyper-immune response also referred as a cytokine storm? Likely yes is the answer.

Some TLR4-activated danger-associated molecular pattern (DAMP) signals, including oxidized 1-palmitoyl-2-arachidonoyl-phosphatidylcholine (OxPAPC) and high-mobility group box 1 (HMGB1), are increased in the acute lung injury (ALI) caused by respiratory viruses such as the influenza virus (125, 126). It is important to note that influenza-triggered ALI seems to occur secondary to the cytokine storm induced by the activation of TLR4 by host-derived DAMPs such as OxPAPC and HMGB1 (125, 127). Notably, TLR4^−/−^ mice have been protected against influenza A virus-provoked lethality, and the therapeutic treatment with TLR4 antagonists, Eritoran and FP7, inhibited influenza virus-induced cytokine production, ALI, and mortality in wild-type mice (127–129).

Interestingly, low doses of LPS exacerbate the TLR3 activation-induced inflammatory response in human monocytes *in vitro* (130). Furthermore, macrophages infected with Influenza A and stimulated with low concentrations of LPS showed increased levels of cytokines compared to macrophages that were infected only with the virus. The authors proposed that LPS enhances the release of bioactive cytokines by infected macrophages, which can lead to a decompensated increase in inflammatory metabolites (131, 132). These data reinforce the idea that weakness of intestinal permeability and consequent translocation of LPS in the elderly, diabetic and hypertensive individuals can be relevant to the severity of COVID-19 in these populations.

In a clinical setting involving 48 subjects, the expression of TLR4 and its downstream signaling molecules as well as S100A9 (TLR4 ligand) were significantly upregulated in PBMCs from severe COVID-19 patients as compared to those from healthy controls. Furthermore, S100A9 amplified the recombinant S2 protein of SARS-CoV-2-induced IL-1β mRNA expression in PBMCs *in vitro* (133), suggesting that activation of TLR4 by LPS from the gut microbiota of elderly, diabetic, and hypertensive individuals may be related to the severity of COVID-19. In keeping with these results, respiratory syncytial virus infection induced an increase of TLR4 expression in the airway epithelial cells *in vitro*, and activation of these cells with LPS potentiated the release of IL-6 and IL-8 induced by the virus (134).

Since severe COVID-19 patients show high expression of TLR4 in PBMCs (133), we can speculate that the activation of this receptor by LPS derived from the gut microbiota of elderly, diabetic, and hypertensive individuals would also potentiate the production of IL-6 induced by SARS-CoV-2. In this respect, it should be pointed out that, among all increased cytokines, the rise of IL-6 circulating levels predicted mechanical ventilation, intensive care unit admission, shock, and death in severe patients with COVID-19 (18, 135, 136). Furthermore, a follow-up with 21 individuals with several or critical COVID-19 revealed that a single dose of tocilizumab, an anti-IL-6 receptor drug, recovered 90% of patients (137).

## Conclusion

**Figure 1:**
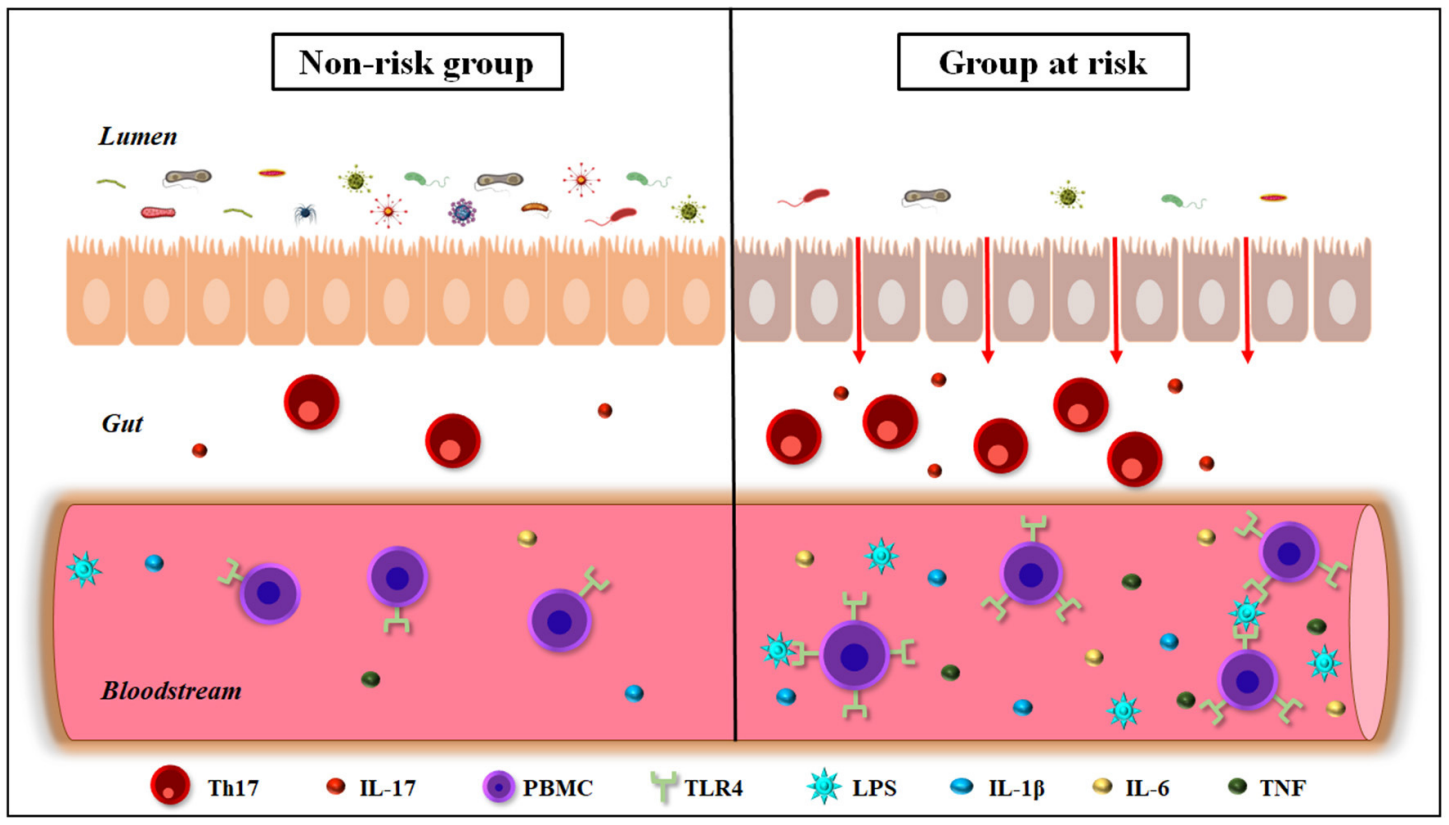
Gut-immune interactions in elderly, diabetic, and hypertensive individuals. These conditions are the three most COVID-19-related death risk factors, and show a decrease in the diversity of the gut microbiota, leading to dysbiosis and weakness of the intestinal barrier permeability. In addition, people belonging to risk groups for COVID-19-related death show hyperimmune activation in the intestine, increasing Th17^+^ T cells and IL-17 production. These individuals also exhibit a rise in the circulating levels of bacterial endotoxins such as LPS, as well as pro-inflammatory cytokines, as IL-1β, IL-6, and TNF. Furthermore, the elderly, diabetic, and hypertensive individuals show an increase in the expression of TLR4 in peripheral blood mononuclear cells (PBMCs). IL-17, interleukin-17; IL-1 β, interleukin-1β; IL-6, interleukin-6; LPS, lipopolysaccharide; Th17, T helper 17; TLR4, Toll-like receptor 4; TNF, Tumor necrosis factor.

In conclusion, we postulate that the gut dysbiosis may be responsible for COVID-19-related death in elderly individuals as well as diabetic and hypertensive patients, since these subjects show a change in the profile of gut microbiota followed by low-grade inflammation, especially with high circulating levels of IL-6. The possibility does exist that augmentation of pro-inflammatory bacteria in the gut may alter the intestinal immune repertoire with consequent weakness of epithelium-intestinal permeability and increased LPS translocation into the bloodstream. We believe that the hyperactivation of TLR4 induced by gut microbiota products, translocated into the circulation, strongly contributes to the cytokine storm, worsening the prognosis of COVID-19 in the elderly, diabetic and hypertensive individuals (Figure 1). In this respect, new therapeutic strategies based on prebiotics or bacterial metabolites, as butyrate, appear as potentially practical approaches for adjuvant treatment of these patients.

## Author Contributions

NM contributed to the conception and design of the study, wrote the manuscript, discussed the content and contributed to the manuscript revision. PS, MM, and WS discussed the content and contributed to the manuscript revision. VC contributed to the conception and design of the study, wrote the manuscript, discussed the content and contributed to the manuscript revision. All authors reviewed and/or edited the manuscript prior submission.

## Conflict of Interest

The authors declare that the research was conducted in the absence of any commercial or financial relationships that could be construed as a potential conflict of interest.

## Publisher's Note

All claims expressed in this article are solely those of the authors and do not necessarily represent those of their affiliated organizations, or those of the publisher, the editors and the reviewers. Any product that may be evaluated in this article, or claim that may be made by its manufacturer, is not guaranteed or endorsed by the publisher.

## Abbreviations

ACE2: angiotensin I converting enzyme 2
ALI: acute lung injury
AMP: anti-microbial peptides
ARDS: acute respiratory distress syndrome
BP: blood pressure
CD3: cluster of differentiation 3
COV: coronavirus
DAMP: danger-associated molecular pattern
DM: diabetes mellitus
ENS: enteric nervous system
GF: germ-free
HMGB1: high-mobility group box 1
IFN-γ: interferon gamma γ
IgA: immunoglobulin A
IL-10: interleukin 10
IL-17: interleukin 17
IL-1β: interleukin 1β
IL-6: interleukin 6
IL-7: interleukin 7
IL-8: interleukin 8
LADA: Latent Autoimmune Diabetes in Adults
Lcp2: lymphocyte cytosolic protein 2
LPS: lipopolysaccharide
mRNA: messenger ribonucleic acid
OxPAPC: oxidized 1-palmitoyl-2-arachidonoyl-phosphatidylcholine
PBMC: peripheral blood mononuclear cells
SARS: severe acute respiratory syndrome
SCFA: short-chain fatty acids
SHR: hypertensive rats
TGF-β1: Transforming growth factor beta 1
Th17: T helper 17
TLR4: toll-like receptor 4
TMA: Trimethylamine
TNF: Tumor necrosis factor
WKY: Wistar Kyoto
WT: wild type.
